# Critical Role of Non-Muscle Myosin Light Chain Kinase in Thrombin-Induced Endothelial Cell Inflammation and Lung PMN Infiltration

**DOI:** 10.1371/journal.pone.0059965

**Published:** 2013-03-21

**Authors:** Fabeha Fazal, Kaiser M. Bijli, Matthew Murrill, Antony Leonard, Mohammad Minhajuddin, Khandaker N. Anwar, Jacob N. Finkelstein, D. Martin Watterson, Arshad Rahman

**Affiliations:** 1 Department of Pediatrics, University of Rochester School of Medicine and Dentistry, Rochester, New York, United States of America; 2 Department of Medicine, University of Illinois College of Medicine, Chicago, Illinois, United States of America; 3 Center for Molecular Innovation and Drug Discovery, Northwestern University, Chicago, Illinois, United States of America; University of Texas Health Science Center at Tyler, United States of America

## Abstract

The pathogenesis of acute lung injury (ALI) involves bidirectional cooperation and close interaction between inflammatory and coagulation pathways. A key molecule linking coagulation and inflammation is the procoagulant thrombin, a serine protease whose concentration is elevated in plasma and lavage fluids of patients with ALI and acute respiratory distress syndrome (ARDS). However, little is known about the mechanism by which thrombin contributes to lung inflammatory response. In this study, we developed a new mouse model that permits investigation of lung inflammation associated with intravascular coagulation. Using this mouse model and *in vitro* approaches, we addressed the role of non-muscle myosin light chain kinase (nmMLCK) in thrombin-induced endothelial cell (EC) inflammation and lung neutrophil (PMN) infiltration. Our *in vitro* experiments revealed a key role of nmMLCK in ICAM-1 expression by its ability to control nuclear translocation and transcriptional capacity of RelA/p65 in EC. When subjected to intraperitoneal thrombin challenge, wild type mice showed a marked increase in lung PMN infiltration via expression of ICAM-1. However, these responses were markedly attenuated in mice deficient in nmMLCK. These results provide mechanistic insight into lung inflammatory response associated with intravascular coagulation and identify nmMLCK as a critical target for modulation of lung inflammation.

## Introduction

A hallmark of acute lung injury (ALI) and its more severe form acute respiratory distress syndrome (ARDS) is an exuberant inflammatory response characterized by massive infiltration of polymorphonuclear lymphocytes (PMN) into the lung that ultimately leads to disruption of capillary-alveolar barriers and development of pulmonary edema with severe consequences for pulmonary gas exchange [1], [2]. An emerging paradigm is that uncontrolled activation of the coagulation cascade after inflammation and tissue injury plays an important role in the pathogenesis of ALI/ARDS [3]–[6]. It is becoming increasingly clear that a close interaction and bidirectional cooperation exists between inflammation and coagulation, particularly in the setting of sepsis (a prominent extrapulmonary cause of ALI responsible for 40% of ALI in humans) [7] whereby inflammation not only triggers activation of coagulation, but coagulation also leads to inflammation [8], [9]. A key molecule linking coagulation and inflammation is thrombin, a procoagulant serine protease whose concentrations are elevated in plasma and lavage fluids of patients with ALI/ARDS [10], [11]. Studies in animal models have shown that infusion of thrombin induces lung vascular injury and tissue edema and that these responses are critically dependent on PMN sequestration in microvessels [12], [13]. However, the mechanism by which thrombin promotes lung PMN sequestration remains poorly understood, largely due to the lack of a mouse model of thrombin-induced lung PMN infiltration.

A critical step in the mechanism of PMN sequestration in the lung involves stable adhesion of PMN to the vascular endothelium, which is mediated by interaction of intercellular adhesion molecule-1 (ICAM-1; CD54) on endothelial cell (EC) surface with its counter-receptor β_2_-integrins LFA-1 (CD11a/CD18) and Mac-1 CD11b/CD18) on PMN [14]–[16]. Interaction of EC with PMN by this mechanism is required for PMN migration across endothelial barrier into the interstitium [16], [17]. More recently, ligation of ICAM-1 has also been shown to induce endothelial permeability [18], suggesting dual function of ICAM-1 in mediating PMN transmigration and EC permeability associated with lung inflammation. We and others have shown that up-regulation of ICAM-1 expression by thrombin depends primarily on activation of the transcription factor NF-κB (predominantly RelA/p65 homodimer) [19] and that this response is mediated through activation of the GTP-binding protein (G-protein) coupled receptor, protease-activated receptor-1 (PAR-1) [19], [20]. In most cases, the initiating event in NF-κB activation involves stimulation of IκBβ kinase (IKKβ) activity which phosphorylates two specific serine residues (Ser^32^ and Ser^36^) of IκBα, an inhibitory protein that retains NF-κB in the cytoplasm. Upon phosphorylation, IκBα undergoes rapid polyubiquitination, which targets it for degradation by the 26S proteasome [21]–[23]. The liberated NF-κB undergoes rapid cytoplasmic trafficking and nuclear import to activate the transcription of target genes, such as ICAM-1 [23]. While the events mediating the release of RelA/p65 from NF-κB are well established, the mechanisms controlling its movement from cytoplasm to the nucleus remain largely unknown.

MLCK is a calcium-calmodulin-dependent kinase dedicated to myosin II regulatory light chain (MLC) [24]. It is expressed as two isoforms; smooth muscle MLCK (108–130 kDa) and nonmuscle MLCK (nmMLCK: 210 kDa) [24], [25], also known as EC-MLCK because of its abundance in the endothelium. Studies have shown that nmMLCK is a key determinant of endothelial barrier disruption through its ability to regulate actomyosin contractility in EC stimulated with edemagenic agonists such as thrombin [26], [27]. Consistent with this, nmMLCK knockout (nmMLCK^−/−^) mice are protected from ventilation- and endotoxin-induced ALI and also show much better survival [28]–[30].

In view of the ability of thrombin to engage MLCK to phosphorylate myosin light chain (MLC) and thus increase actin-myosin interaction [26], [27] and our recent finding that actin cytoskeleton reorganization is critical for ICAM-1 expression [31], [32], we addressed the role of nmMLCK in the mechanism of thrombin-induced RelA/p65 activation and EC inflammation. We also report the development of a new *in vivo* mouse model that permits investigation of acute lung inflammation associated with intravascular coagulation. Using integrated *in vitro* and *in vivo* approaches, we show an important role of nmMLCK in causing EC inflammation by its ability to mediate nuclear translocation and transcriptional capacity of RelA/p65, and consequently, PMN infiltration in the lungs of mice challenged with thrombin.

## Materials and Methods

### Ethics Statement

All mice care and treatment procedures were performed in adherence to the National Institute of Health guidelines and approved by the University of Rochester Committee on Animal Resources (PHS Animal Welfare Assurance Number A329201).

### Reagents

Human thrombin was obtained from Enzyme Research Laboratories (South Bend, IN). ML-7 was purchased from Calbiochem-Novabiochem Corp. (La Jolla, CA). Polyclonal antibodies to ICAM-1, nmMLCK, RelA/p65, β-actin, IκBα, Cu-Zn superoxide dismutase (SOD-1), and TATA-binding protein (TBP) were from Santa Cruz Biotechnology (Santa Cruz, CA). Antibodies to phospho-(Ser32 and Ser36)-IκBα, phospho-(Thr180/Tyr182)-p38 MAP kinase, phospho-(Ser536)-RelA/p65, were obtained from Cell Signaling (Beverly, MA). RelA/p65 transcription factor assay kit was purchased from Cayman Chemical (Ann Arbor, MI) and plasmid maxi kit was from QIAGEN Inc. (Valencia, CA). All other materials were from VWR Scientific Products Corporation (Gaithersburg, MD) and Fisher Scientific (Pittsburgh, PA).

### Cell Culture

Human umbilical vein endothelial cell (HUVEC) cultures were established by using umbilical cords collected within 48 h of delivery. Human pulmonary artery endothelial cells (HPAEC) were purchased from Lonza (Walkersville, MD). Cells were cultured as described previously [31], [33] in endothelial basal medium 2 (EBM2) supplemented with bullet kit^tm^ additives (BioWhittaker, Walkersville, MD) and were used between passages 3 and 6.

### Mice

Generation of nmMLCK−/− mice (KO) on C57BL/6 background was described previously [28]. The C57BL/6 wild-type (WT) mice were obtained from Jackson Laboratory (Bar Harbor, ME). Male mice (8–12 weeks of age) were matched in accordance with the weight, which ranged between 20–25 g. In some experiments both male and female mice were used.

### Murine Model of Thrombin-Induced Lung Inflammation

Lung inflammation was induced by intraperitoneal (i.p.) injection of thrombin (50–70 units/g body weight). Lungs from WT and KO mice were then collected at the indicated times after thrombin administration and analyzed for expression of ICAM-1 and monocyte chemoattractant protein 1 (MCP-1), histological examination, and lung PMN infiltration. MLCK inhibitor (ML-7; 2.5 mg/kg) was administered to WT mice by i.p. injection 0.5 h prior to treatment with thrombin.

### RNAi Knockdown

SMARTpool siRNA specific for human nmMLCK and a non-targeting siRNA control were purchased from Dharmacon (Lafayette, CO). EC were transfected with nmMLCK or control siRNA using DharmaFect1 siRNA Transfection Reagent (Dharmacon) essentially as described [32].

### NF-κB Transcriptional Activity

The transcriptional activity of NF-κB was measured as described [32]. EC were transfected with the plasmid NF-κB-LUC containing 5 copies of consensus NF-κB sequences linked to a minimal E1B promoter-firefly luciferase gene using DEAE-dextran [32]. The plasmid TKRLUC (Promega, Madison, WI) containing *Renilla* luciferase reporter gene driven by constitutively active thymidine kinase promoter was used to normalize the transfection efficiency. In experiments evaluating the effect of nmMLCK knockdown on NF-κB transcriptional activity, cells were first transfected with nmMLCK siRNA as described above. After 24 h, cells were again transfected with NF-κB-LUC and TKRLUC plasmids using DEAE-dextran essentially as described [32]. After appropriate treatments, cell extracts were prepared and assayed for Firefly and *Renilla* luciferase activities using Promega Biotech Dual Luciferase Reporter Assay System. The data were expressed as a ratio of Firefly to *Renilla* (F/R) luciferase activity.

### Northern Analysis

Total RNA was isolated using RNeasy kit (QIAGEN Inc.) according to manufacturer’s recommendations. Quantification and purity of RNA were assessed by A_260_/A_280_ absorption and an aliquot of RNA (20 µg) from samples with ratio above 1.6 was subjected to Northern blot analysis as described [34].

### Immunoblot Analysis

Cells were lysed in phosphorylation lysis buffer (50 mM HEPES, 150 mM NaCl, 200 µM sodium orthovanadate, 10 mM sodium pyrophosphate, 100 mM sodium fluoride, 1 mM EDTA, 1.5 mM magnesium chloride, 10% glycerol, 0.5 to 1% Triton X-100, 1 mM phenylmethylsulfonyl fluoride [PMSF], and protease inhibitor cocktail [Sigma-Aldrich, St. Louis, MO]) or in radioimmune precipitation (RIPA) buffer (50 mM Tris-HCl, pH 7.4, 150 mM NaCl, 0.25 mM EDTA, pH 8.0, 1% deoxycholic acid, 1% Triton-X, 5 mM NaF, 1 mM sodium orthovanadate supplemented with protease inhibitor cocktail). Lung samples were also homogenized in RIPA buffer supplemented with protease inhibitor cocktail. Briefly, lung tissue (100 mg) was mechanically homogenized in 0.5 ml of RIPA buffer, and the homogenates were incubated on ice for 1 h to achieve total cell lysis. Equal amounts of protein from the cell lysates or lung homogenates were electrophoresed and subsequently transferred onto nitrocellulose membranes. The membranes were blocked with 5% (w/v) nonfat dry milk and immunoblotted with appropriate antibody as described [32], [35]. Representative blots presented in the results section come from the same membrane which may have more samples in various groups.

### ELISA

The levels of MCP-1 in HPAEC culture supernatants and in mouse lung homogenates were determined using ELISA kits from R&D Systems (Minneapolis, MN) and Millipore Corp. (Bradford, MA) according to manufacturers’ recommendations.

### Nuclear Extract Preparation and Assessment of RelA/p65 DNA Binding

After treatment, cells were washed twice with ice-cold phosphate-buffered saline and resuspended in 400 µl of buffer A (10 mM HEPES [pH 7.9], 10 mM KCl, 0.1 mM EDTA, 0.1 mM EGTA, 1 mM [DTT], and 0.5 mM PMSF). Fifteen minutes later, NP-40 was added to a final concentration of 0.6%, and the samples were centrifuged to collect the supernatants containing the cytoplasmic proteins. The pelleted nuclei were resuspended in 50 µl of buffer B (20 mM HEPES [pH 7.9], 0.4 M NaCl, 1 mM EDTA, 1 mM EGTA, 1 mM DTT, and 1 mM PMSF). After 0.5 h at 4°C, lysates were centrifuged and supernatants containing the nuclear proteins were collected. The DNA binding activity of RelA/p65 was determined using an ELISA-based DNA binding assay kit (Cayman Chemical, Ann Arbor, MI) in accordance with the manufacturer’s recommendations or by electrophoretic mobility shift assay (EMSA) as described [34].

### Lung Tissue Myeloperoxidase (MPO) Activity

PMN infiltration was quantified by measuring the lung tissue MPO activity as described previously [36]. Briefly, lung tissue (100 mg) was suspended in 1 ml buffer containing 0.5% hexadecyltrimethylammonium bromide in 50 mM phosphate buffer at pH 6.0 and sonicated at 30 cycles, twice, for 30 s on ice [36]. The homogenates were centrifuged at 12,000 rpm for at 4°C, and clear supernatants were collected and stored at −70°C. The protein concentration of the supernatants was determined using a protein quantitation kit from Pierce (Thermofisher Scientific, Rockford, IL). To measure MPO activity, reaction was performed in a 96-well plate by addition of 290 µl of 50 mM phosphate buffer, 3 µl of substrate solution containing 20 mg/ml o-dianisidine hydrochloride, 3 µl of o-dianisidine hydrochloride, and 3 µl of 20 mM H_2_O_2_. Reaction was started by adding supernatant (10µl) to each well. Standard MPO (Sigma Chemical Company, St. Louis, MO) was used to determine the MPO activity in the sample. The reaction was stopped by addition of 3 µl of 30% sodium azide. Samples were read at 460 nm. MPO activity was determined by using the curve obtained from the standard MPO (Sigma Chemical Company) and expressed as units/mg protein.

### Histological Analysis

Lungs were inflated and fixed with 10% neutral buffered formalin. Tissues were embedded in paraffin, sectioned (5 µM), and stained with hematoxylin and eosin (H&E).

### Statistical Analysis

**Figure 1:**
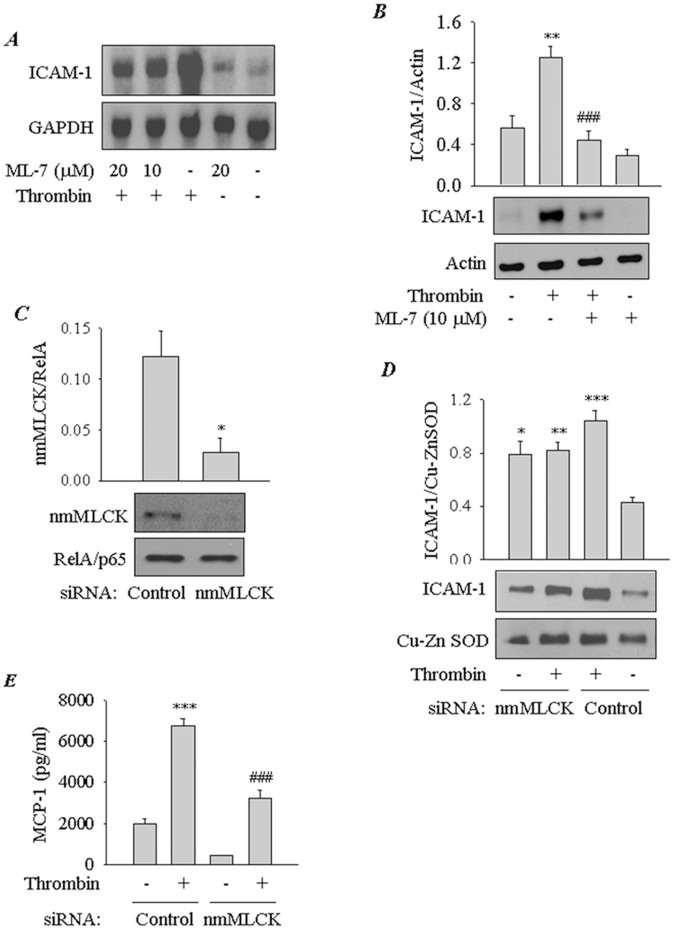
Involvement of nmMLCK in thrombin-induced EC inflammation. *(* ***A*** *)* Effect of inhibition of nmMLCK on thrombin-induced ICAM-1 mRNA expression. Confluent EC monolayers were treated with indicated concentrations of ML-7 prior to challenge with thrombin (5 U/ml) for 3 h. Total RNA was isolated and subjected to Northern blot analysis using a human ICAM-1 cDNA, which hybridizes to a 3.3-kb transcript. GAPDH mRNA levels were determined as a measure of RNA loading. *(* ***B*** *)* Effect of inhibition of nmMLCK on thrombin-induced ICAM-1 protein expression. Confluent EC monolayers were pretreated with ML-7 at the indicated concentration and then challenged with thrombin (5 U/ml) for 6 h. Total cell lysates were separated by SDS-PAGE and immunoblotted with an antibody to ICAM-1. Actin levels were used to monitor loading. The bar graph represents the effect of nmMLCK inhibition on ICAM-1 protein expression normalized to actin level. Data are means±S.E. (*n = *4–5 for each condition). ^**^, *p*<0.01 compared with untreated controls; ^###^, *p*<0.001 compared with thrombin-stimulated controls. *(* ***C***) RNAi knockdown of nmMLCK in EC. Cells were transfected with nmMLCK or control siRNA for 24–36 h as described in Materials and Methods. Total cell lysates were immunoblotted with an antibody to nmMLCK or RelA/p65. The bar graph represents the level of nmMLCK normalized to RelA/p65 level in cells transfected with control or nmMLCK siRNA. Data are mean±S.E. (*n* = 3–4 for each condition) ^*^, *p*<0.05 compared with cells transfected with control siRNA. *(* ***D*** *)* Effect of RNAi knockdown of nmMLCK on thrombin-induced ICAM-1 protein expression. EC were transfected with control or nmMLCK siRNA for 24–36 h as described in Materials and Methods. Cells were challenged with thrombin (5 U/ml) for 6 h. Total cell lysates were immunoblotted with an antibody to ICAM-1. Cu-Zn superoxide dismutase (SOD-1) levels were determined to monitor loading. The bar graph represents the effect of nmMLCK knockdown on ICAM-1 protein expression normalized to SOD1 level. Data are means±S.E. (*n = *3–4 for each condition). ^*^, *p*<0.05; ^**^, *p*<0.01; or ^***^, *p*<0.001 compared with untreated controls; *(* ***E*** *)* Effect of RNAi knockdown of nmMLCK on thrombin-induced MCP-1 release. EC were transfected with control or nmMLCK siRNA for 24–36 h. Cells were challenged with thrombin (5 U/ml) for 6 h and the conditioned media were subjected to ELISA to determine the levels of MCP-1. Data are means±S.E. (*n* = 6–9 for each condition). ^***^, *p*<0.001 compared with untreated control; ^###^, *p*<0.001 compared with thrombin-stimulated control.

Results are presented as mean ± SE and were analyzed by using standard one-way ANOVA. The significance between the groups was determined using Tukey’s test (Prism 5.0, GraphPad Software, San Diego). In some cases (Figs. 1C & 3A), Student’s *t* test was used for comparisons between experimental groups. A *p* value <0.05 between two groups was considered statistically significant. The number of animals used for each experimental group ranged from 3–6 mice.

## Results

### Inhibition or Depletion of nmMLCK Attenuates Thrombin-induced EC Inflammation

To ascertain the role of nmMLCK in the mechanism of EC inflammation, we first examined the effect of ML-7, a relatively specific inhibitor of MLCK, on thrombin-induced expression of ICAM-1. Pretreatment of cells with ML-7 attenuated both ICAM-1 mRNA and protein expression (Fig. 1 A&B). To confirm the involvement of nmMLCK in this response, we used RNAi knockdown approach to selectively deplete nmMLCK in EC. Cells were transfected with siRNA for nmMLCK or control siRNA, and the levels of nmMLCK were determined by immunoblotting. We observed a substantial depletion of nmMLCK in cells transfected with nmMLCK siRNA compared to the cells transfected with control siRNA (Figure 1C). Similar conditions were, therefore, used in subsequent experiments involving siRNA-mediated depletion of nmMLCK. Depletion of nmMLCK by this approach was effective in reducing thrombin-induced ICAM-1 protein expression, despite its ability to increase the basal ICAM-1 levels (Fig. 1D). We also determined the effect of nmMLCK knockdown on monocyte chemoattractant protein (MCP-1)/CC-chemokine ligand 2(CCL2), another thrombin responsive proinflammatory gene in EC [37]. A marked decrease in both basal and thrombin-induced MCP-1 levels was observed in cells transfected with nmMLCK siRNA (Fig. 1E). These data establish an important role of nmMLCK in causing EC inflammation.

### Inhibition or Depletion of nmMLCK Impairs Thrombin-induced RelA/p65 Phosphorylation and Nuclear Translocation without Affecting IκBα Degradation

**Figure 2:**
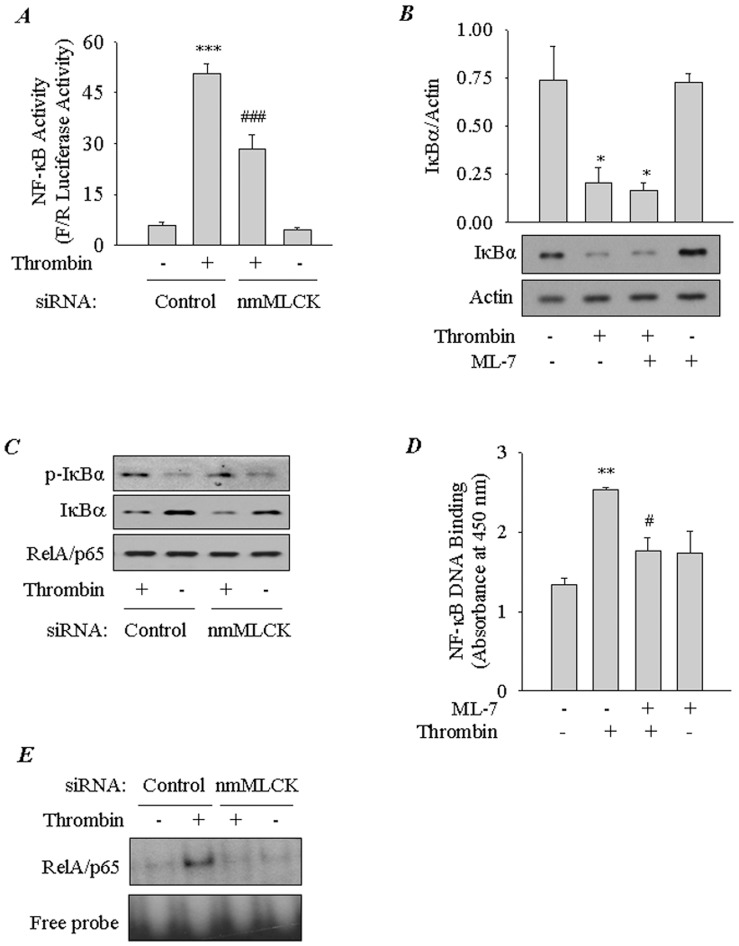
Role of nmMLCK in thrombin-induced NF-κB activation. *(* ***A*** *)* Effect of RNAi knockdown of nmMLCK on thrombin-induced NF-κB transcriptional activity. EC were transfected with control or nmMLCK siRNA using DharmaFect1. Twenty-four hours later, cells were again transfected with NF-κBLUC construct using DEAE-Dextran as described in Materials and Methods. Cells were challenged with thrombin (5 U/ml) for 6 h. Cell extracts were prepared and assayed for Firefly (F) and *Renilla* (R) luciferase activities. Data are means±S.E. (*n = *4 for each condition). ^***^, *p*<0.001 compared with untreated controls; ^###^, *p*<0.001 compared with thrombin-stimulated controls. *(* ***B*** *)* Effect of inhibition of nmMLCK on thrombin-induced IκBα degradation. Confluent EC monolayers were pretreated with ML-7 (10 µM) and then challenged with thrombin (5 U/ml) for 1 h. Total cell lysates were immunoblotted with an anti-IκBαantibody. Actin levels were used to monitor loading. The bar graph represents the effect of inhibition of nmMLCK on thrombin-induced IκBα degradation normalized to actin level. Data are means±S.E. (*n = *3–4 for each condition). ^*^, *p*<0.05 compared with untreated control or ML-7-treated control. *(* ***C*** *)* Effect of nmMLCK knockdown on thrombin-induced IκBα phosphorylation and degradation. EC were transfected with control or nmMLCK siRNA using DharmaFect1. After 24–36 h, cells were challenged for 1 h with thrombin (5 U/ml). Total cell lysates were prepared and immunoblotted with an anti-phospho IκBα (Ser32 and Ser36) or anti-IκBαantibody. RelA/p65 levels were used to monitor loading. The blot is representative of 2 separate experiments. *(* ***D*** *)* Effect of inhibition of nmMLCK on thrombin-induced DNA binding of RelA/p65. EC were pretreated with ML-7 (10 mM) prior to challenge with thrombin for 1 h. Nuclear extracts were prepared and assayed for RelA/p65 DNA binding activity by ELISA as described in Materials and Methods. Data are means±S.E. (*n = *3 for each condition). ^**^, *p*<0.01 compared with untreated control; ^#^, *p*<0.05 compared with thrombin-stimulated control. *(* ***E*** *)* Effect of nmMLCK knockdown on thrombin-induced DNA binding of RelA/p65. EC were transfected with control or nmMLCK siRNA for 24–36 h as described in Materials and Methods. Cells were challenged with thrombin (5 U/ml) for 1 h. Nuclear extracts were prepared and assayed for RelA/p65 DNA binding activity by EMSA as described in Materials and Methods. Representative of two experiments.

**Figure 3:**
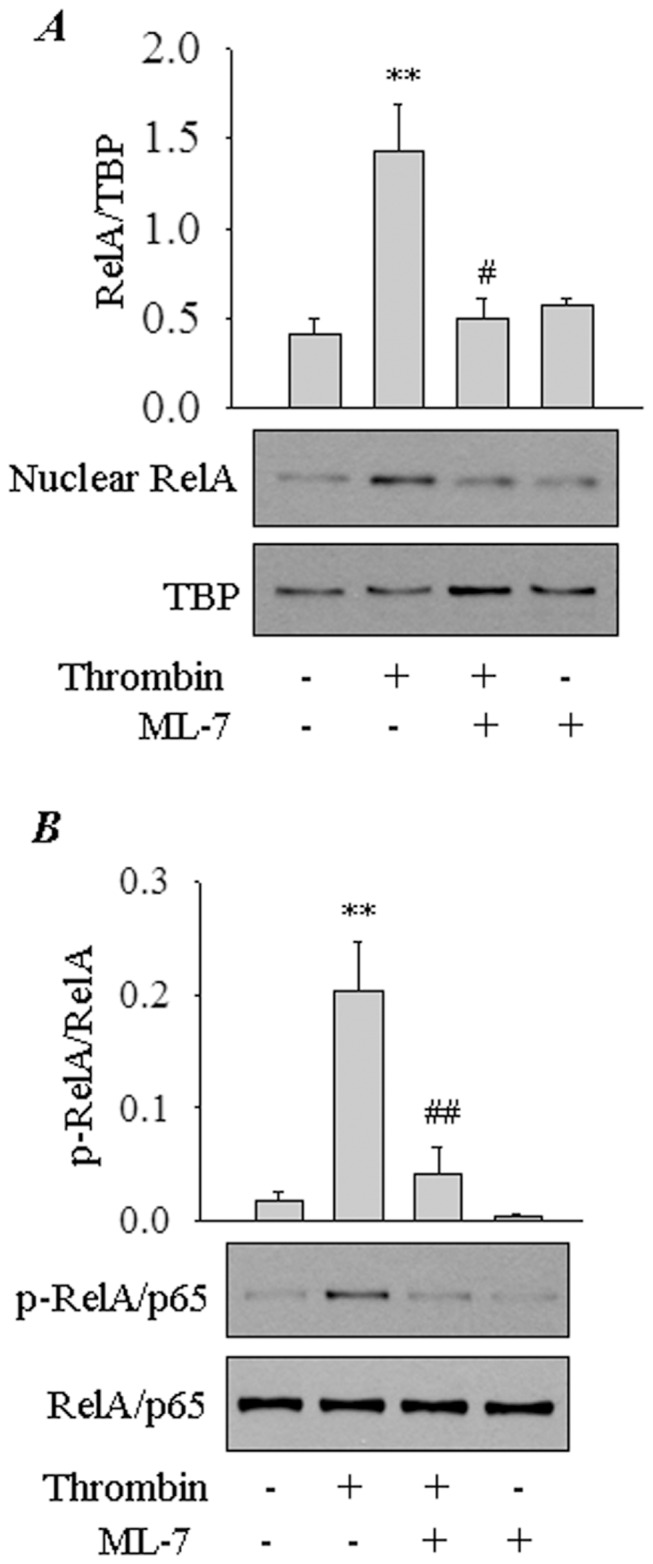
Role of nmMLCK in thrombin-induced RelA/p65 nuclear translocation and phosphorylation. *(* ***A*** *)* Effect of inhibition of nmMLCK on thrombin-induced nuclear translocation of RelA/p65. EC were pretreated with ML-7 (10 µM) and then challenged with thrombin (5 U/ml) for 1 h. Nuclear extracts were separated by SDS-PAGE and immunoblotted with anti-RelA/p65 antibody. TATA-binding protein (TBP) levels were used to monitor loading. The bar graph represents the effect of nmMLCK inhibition on thrombin-induced RelA/p65 nuclear translocation. Data are means±S.E. (*n = *3–4 for each condition). ^**^, *p*<0.01 compared with untreated control; ^#^, *p*<0.05 compared with thrombin-stimulated control. *(* ***B*** *)* Effect of inhibition of nmMLCK on thrombin-induced phosphorylation of RelA/p65. EC were pretreated with ML-7 (10 µM) and then challenged with thrombin (5 U/ml) for 1 h. Total cell lysates were immunoblotted with an anti-phospho-RelA/p65 (Ser536) antibody. RelA/p65 levels were used to monitor loading. The bar graph represents the effect of nmMLCK inhibition on thrombin-induced RelA/p65 phosphorylation. Data are means±S.E. (*n = *3 for each condition). ^**^, *p*<0.01 compared with untreated control; ^##^, *p*<0.01 compared with thrombin-stimulated control.

We next asked if nmMLCK mediates EC inflammation by promoting the activity of NF-κB, a critical regulator of genes encoding ICAM-1 and MCP-1 [19], [38]. Result showed that knockdown of nmMLCK attenuated NF-κB-dependent luciferase activity induced by thrombin (Fig. 2A). To determine how nmMLCK regulates NF-κB activity, we analyzed the effect of inhibiting or depleting nmMLCK on NF-κB signaling pathway. Inhibition or depletion of nmMLCK, each failed to prevent thrombin-induced IκBα phosphorylation and degradation, a requirement for the release of RelA/p65 for its nuclear translocation (Fig. 2B&C and data not shown). We next determined the effect of inhibiting nmMLCK on NF-κB DNA binding activity. We have previously established that thrombin-induced NF-κB complexes are predominantly composed of RelA/p65 homodimer [19], [31]. Surprisingly, in contrast to its effect on IκBα degradation, nmMLCK inhibition or depletion was associated with a marked reduction in thrombin-induced RelA/p65 DNA binding in the nucleus (Fig. 2D&E). These data led us to test the possibility that nmMLCK inhibition interferes with translocation of the released RelA/p65 to the nucleus. Consistent with this possibility, RelA/p65 nuclear localization in response to thrombin was attenuated in cells pretreated with ML-7 (Fig. 3A). Because Ser536 phosphorylation of RelA/p65 promotes the transcriptional activity of RelA/p65 bound to the DNA [23], we examined the role of nmMLCK in mediating this response. We found that thrombin-induced RelA/p65 phosphorylation was lost upon nmMLCK inhibition (Fig. 3B). Together, these data implicate a role for MLCK in causing EC inflammation by facilitating the nuclear uptake and enhancing the transcriptional capacity of RelA/p65.

### Inhibition or Deletion of nmMLCK Attenuates Thrombin-induced Lung PMN Infiltration

**Figure 4:**
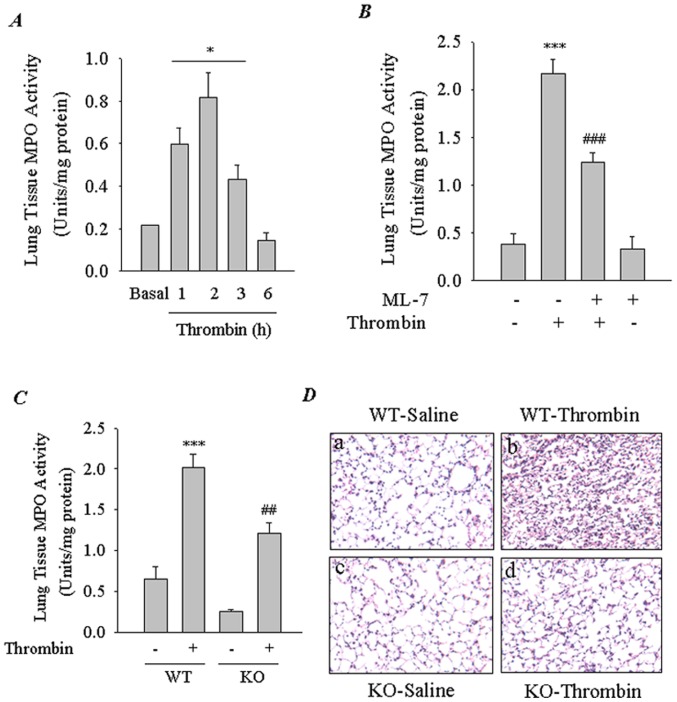
Role of nmMLCK in thrombin-induced lung PMN infiltration. *(* ***A*** *)* Time course of thrombin-induced lung PMN infiltration. Mice were challenged i.p. with thrombin for indicated times. Lungs were isolated and analyzed for PMN infiltration by measuring tissue MPO activity. Bars indicate mean ± SEM (n = 3–6 for each condition). ^*^Different from untreated (basal) control (*p*<0.05). *(* ***B*** *)* Effect of inhibition of nmMLCK on thrombin-induced lung PMN infiltration. Mice were injected i.p. with ML-7 (2.5 mg/kg) 0.5 h before thrombin challenge of mice. After 2 h, lungs were isolated and analyzed for PMN infiltration by measuring tissue MPO activity. Data are mean ± SEM (*n* = 4–6 for each condition). ^***^, *p*<0.001 compared with unchallenged control; ^###^, *p*<0.001 compared with thrombin-challenged control. *(* ***C*** *)* Effect of nmMLCK deficiency on thrombin-induced lung PMN infiltration. Wild-type (WT) and nmMLCK knock out (KO) mice were challenged with thrombin. After 2.5 h, lungs were isolated and analyzed for PMN infiltration by measuring tissue MPO activity. Data are mean ± SEM (*n* = 3–5 for each condition). ^***^, *p*<0.001 compared with unchallenged WT control; ^##^, *p*<0.01 compared with thrombin-challenged WT control. *(* ***D*** *)* Hematoxylin/eosin (H&E) staining of lung sections from WT and nmMLCK knockout (KO) mice 2 h after after i.p. injection of thrombin. Representative images are shown. n = 3–4 mice for each experimental group.

To determine if the above effects of nmMLCK inhibition can be recapitulated *in vivo* in the intact lungs of mice, we developed an *in vivo* mouse model of lung PMN sequestration associated with intravascular coagulation. Mice were challenged i.p. with thrombin for indicated times, and the lungs were analyzed for PMN infiltration by measuring MPO activity. Thrombin induced lung PMN infiltration in a time-dependent manner with maximal response occurring at 2 h after thrombin challenge (Fig. 4A). We used this mouse model to evaluate the role of nmMLCK in the mechanism of lung PMN infiltration. Mice were exposed to ML-7 prior to challenge with thrombin. Lung tissue MPO activity increased ∼ 4 fold after thrombin challenge. Exposure of mice to ML-7 reduced MPO activity by ∼ 50% of the control values (Fig. 4B). To verify these data, mice with targeted deletion of nmMLCK were challenged with thrombin and the lung tissues from these mice were analyzed for MPO activity. Results showed that both basal and thrombin-induced MPO activity were attenuated in nmMLCK-deficient mice (Fig. 4C), confirming the involvement of nmMLCK in lung PMN infiltration. We also performed histological examination of the lung tissue from WT and KO mice to further verify the role of nmMLCK in lung inflammatory responses. In WT mice exposed to thrombin, we found evidence of inflammatory cell infiltrate, intravascular and interstitial hemorrhage, and atelectasis (Fig. 4D; panel b). By contrast, in KO mice exposed to thrombin, these markers of lung vascular inflammation and permeability were drastically reduced (Fig. 4D; panel d). These data indicate that the barrier integrity of the endothelium in the KO lung is not disrupted by thrombin, consistent with the established role of nmMLCK in endothelial barrier dysfunction [26], [27].

### Blockade of ICAM-1 Reduces Thrombin-induced Lung PMN Infiltration

**Figure 5:**
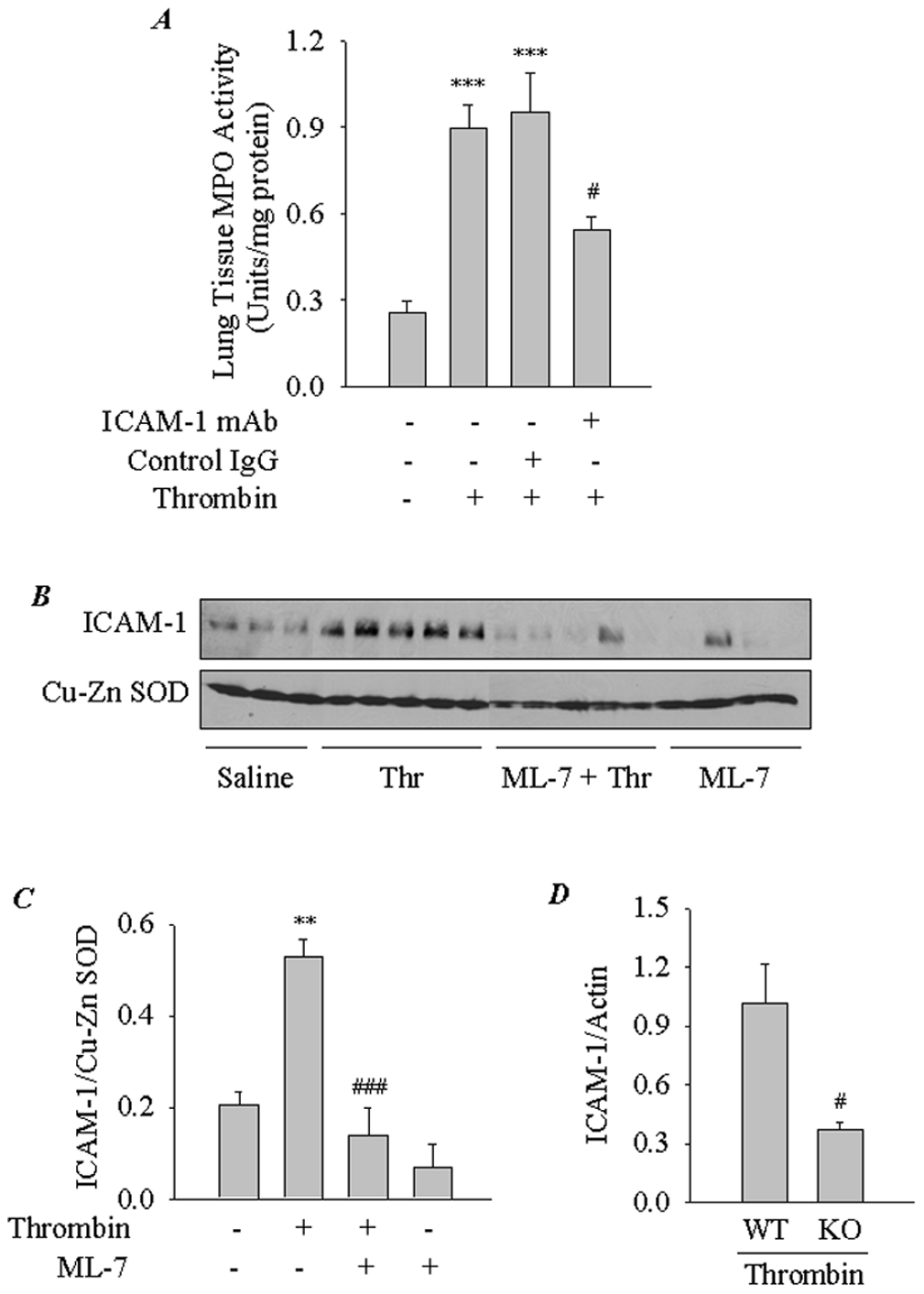
Involvement of ICAM-1 in thrombin-induced lung PMN infiltration. *(* ***A*** *)* Effect of ICAM-1 blockade on thrombin-induced lung PMN infiltration in mice. ICAM-1 mAb (1 mg/kg body weight) or rat IgG was injected i.p. 0.5 h before thrombin challenge of mice. After 2 h, lungs were isolated and analyzed for PMN infiltration by measuring MPO activity. Bars indicate mean ± SEM (n = 5–6 for each condition). ^***^, *p*<0.001 compared with unchallenged control; ^##^, *p*<0.01 compared with thrombin-challenged control or thrombin-challenged IgG control. *(* ***B*** *)* Effect of ML-7 on thrombin-induced ICAM-1 expression in the lungs of mice. ML-7 (2.5 mg/kg) was injected i.p. 0.5 h before thrombin challenge of mice. After 2 h, lungs were isolated and analyzed for ICAM-1 expression by immunoblotting. Cu-Zn SOD levels were used to monitor loading. *(* ***C*** *)* The bar graph represents the effect of ML-7 on thrombin-induced ICAM-1 expression in *(* ***B*** *).* ICAM-1 protein expression normalized to Cu-Zn SOD level is expressed as ICAM-1/Cu-Zn SOD ratio. Data are mean ± SEM (*n* = 3–5 for each condition). ^**^, *p*<0.01 compared with unchallenged control; ^##^, *p*<0.001 compared with thrombin-challenged control. *(* ***D*** *)* The bar graph represents the effect of nmMLCK deficiency on ICAM-1 expression in lungs of mice challenged with thrombin. WT and nmMLCK−/− mice (KO) mice were injected i.p. with thrombin. After 2 h, lungs were isolated and analyzed for ICAM-1 expression by immunoblotting. Actin levels were used to monitor loading. ICAM-1 protein expression normalized to actin level is expressed as ICAM-1/Actin ratio. Data are mean ± SEM (*n* = 3 for each condition). ^#^, *p*<0.05 compared with thrombin-challenged WT control.

**Figure 6:**
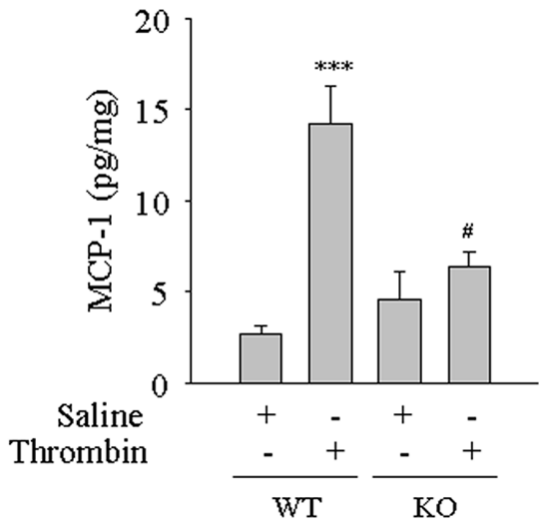
Effect of nmMLCK deficiency on MCP-1 levels in lungs of mice challenged with thrombin. WT and nmMLCK knockout (KO) mice were injected i.p. with thrombin. After 2 h, lungs were isolated and analyzed for MCP-1 expression by ELISA. Data are mean ± SEM (*n* = 3–4 for each condition). ^***^, *p*<0.001 compared with saline-exposed WT control; ^#^, *p*<0.05 compared with thrombin-challenged WT control.

Because ICAM-1 plays an important role in transendothelial migration of leukocytes [14]–[17], we determined if nmMLCK controls lung PMN infiltration via an ICAM-1-dependent mechanism. To this end, studies were made using anti-ICAM-1 blocking antibody in mice challenged with thrombin. Results showed that ICAM-1 blockade induced by ICAM-1 mAb injection reduced lung MPO activity by ∼45% of the control values (Fig. 5A). In parallel experiments, control Ab failed to prevent PMN infiltration in lungs of thrombin-challenged mice (Fig. 5A). These results show *in vivo* contribution of ICAM-1 in lung PMN infiltration induced by thrombin. These data prompted us to address the role of nmMLCK in regulating ICAM-1 levels in the lung. Western blot analysis of lung homogenates from WT mice challenged with thrombin showed increased levels of ICAM-1 and this response was inhibited in mice exposed to ML-7 (Fig. 5B&C) or deficient in nmMLCK (Fig. 5D). We also evaluated the role of nmMLCK in regulating the levels of MCP-1, another thrombin responsive gene implicated in PMN infiltration, in the lungs of mice challenged with thrombin. In KO mice exposed to thrombin, the levels of MCP-1 were significantly reduced compared to WT mice challenged with thrombin (Fig. 6). These results are consistent with the ability of nmMLCK in regulating ICAM-1 and MCP-1 expression in EC (Fig. 1E).

## Discussion

There is clear evidence supporting an essential role of nmMLCK in the regulation of EC barrier function [26], [27]. nmMLCK also plays a causal role in endotoxin-induced lung injury in mice [28]–[30]. Recently, Xu et al. [30] have shown a novel role of nmMLCK in activating β_2_ integrins to promote PMN transmigration in a sepsis-induced mouse model of lung inflammation. However, the role of nmMLCK in mediating the expression of ICAM-1, the counter receptor of β_2_ integrins on EC, and the contribution of nmMLCK/ICAM-1 axis in endothelial regulation of PMN trafficking in the lung, particularly in the setting of intravascular coagulation remains uncertain. In this study, we used integrated *in vitro* and *in vivo* approaches and developed an i.p. thrombin challenge mouse model to investigate the mechanistic basis of nmMLCK signaling to ICAM-1 in EC and the contribution of nmMLCK/ICAM-1 axis in acute lung inflammation associated with intravascular coagulation. Our *in vitro* data establish nmMLCK as an important mediator of ICAM-1 expression by its ability to control the nuclear translocation and transcriptional activity of RelA/p65. Our *in vivo* results show that inhibition or deletion of nmMLCK reduces lung inflammatory responses in mice challenged with thrombin. Similarly, blockade of ICAM-1 also caused a marked reduction in thrombin-induced lung PMN sequestration in mice. Together, these data identify nmMLCK as a critical target for modulation of ALI associated with intravascular coagulation.

**Figure 7:**
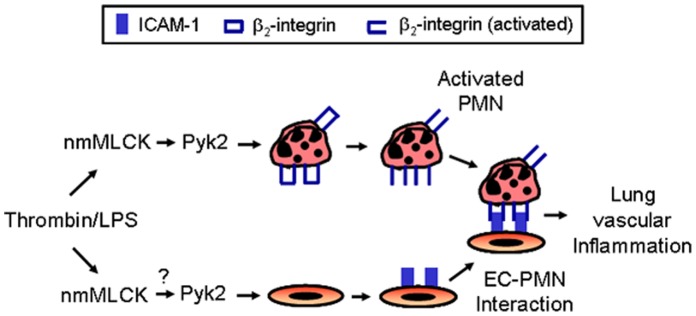
Proposed model for nmMLCK regulation of EC-PMN interactions mediating lung PMN infiltration associated with intravascular coagulation and sepsis. Induction of nmMLCK activity during sepsis leads to activation of Pyk2 which in turn activates β2 integrins in PMN [30]. We propose that nmMLCK activated during intravascular coagulation and/or sepsis also engages Pyk2 in EC to induce ICAM-1 expression. Activation of β2 integrins and expression of ICAM-1 by this mechanism serves to promote EC-PMN interactions and lung vascular injury associated with intravascular coagulation and sepsis.

Studies have shown that endothelial ICAM-1 plays an important role in lung PMN recruitment via its interaction with β_2_ integrin LFA-1 on PMN [14], [16], [17]. Mice deficient in ICAM-1 or LFA-1 each showed reduced PMN recruitment (∼50%) into the lung/alveolar space induced by LPS inhalation [39]. Similarly, blockade of ICAM-1 or LFA-1 was associated with marked reduction in lung PMN recruitment and lung vascular permeability in wild-type mice when exposed to LPS or *live E.* coli [39]–[42]. Intriguingly, ligation of endothelial ICAM-1 by PMN also contributes to pulmonary vascular permeability via caveolin-1 and caveolae-mediated transcytosis or RhoA-mediated EC barrier dysfunction [18], [43]. Thus, our findings that nmMLCK controls ICAM-1 expression in EC *in vitro* and in the lung *in vivo* and that the blockade of ICAM-1 causes a marked reduction in thrombin-induced lung PMN sequestration in mice underscores the importance of endothelial nmMLCK/ICAM-1 axis in the mechanism of lung vascular inflammation. Taking into account the studies by Xu et al. [30] showing a pivotal role of nmMLCK in activating β_2_ integrins to promote sepsis-induced lung PMN recruitment in mice, our results are consistent with a model (Fig. 7) wherein nmMLCK activity in EC and PMN induce the expression of ICAM-1 and activation of β_2_ integrins, respectively to promote EC-PMN interactions and the associated lung vascular inflammation and permeability.

An essential event mediating the expression of ICAM-1, MCP-1 and other proinflammatory genes in the endothelium involves activation of NF-κB [23], [44]. Notably, EC-selective blockade of NF-κB activation has been shown to inhibit adhesion molecule expression, attenuate lung inflammation, prevent vascular leak, and improve survival in murine models of sepsis [45], indicating the cardinal role played by endothelial NF-κB in the pathogenesis of ALI. Our data showing that inhibition or depletion of nmMLCK reduces NF-κB activation is consistent with a role of nmMLCK in regulating ICAM-1 via NF-κB. Similarly, a role of nmMLCK in LPS-induced NF-κB in has also been reported [46], [47]. Further analysis of NF-κB signaling pathway showed that nmMLCK controls NF-κB activity by facilitating the translocation of RelA/p65 to the nucleus. In addition to promoting nuclear translocation, nmMLCK also increases the transcriptional capacity of RelA/p65 through its phosphorylation at Ser536. This mechanism of NF-κB activation is strikingly similar to our earlier findings that activation of RhoA/ROCK/Cofilin1 pathway and the associated changes in actin cytoskeleton are necessary for thrombin-induced RelA/p65 nuclear translocation and thereby ICAM-1 expression [31], [32]. Interestingly, this (RhoA/ROCK/Cofilin1) pathway is not restricted to RelA/p65 nuclear translocation, but is also utilized by shear stress to activate sterol regulatory element-binding proteins in EC [48]. Because nmMLCK is a major actin binding protein and regulator of the actin cytoskeleton reorganization, our results point to the possibility that nmMLCK contributes to RelA/p65 nuclear translocation and thereby ICAM-1 expression by inducing actin-myosin interaction (actin stress fiber formation). Given that thrombin engages nmMLCK to catalyze MLC phosphorylation [24], [49], it is likely that the putative transporting system mediating RelA/p65 transport toward the nucleus may involve motor proteins such as myosin. Such a possibility is further supported by our findings that inhibition of myosin prevents thrombin-induced ICAM-1 expression [32].

Another mechanism by which nmMLCK can contribute to regulation of RelA/p65 and ICAM-1 expression may involve its scaffolding function. It should be noted that nmMLCK, in addition to its kinase domain, harbors sites that enable it to interact with and regulate cytoskeletal proteins and tyrosine kinases [50]. In the context of the present study, tyrosine kinase Pyk2 is of particular interest among the proteins interacting with nmMLCK. Xu et al. [30] have recently shown that nmMLCK stimulates Pyk2, in part, through its scaffolding function to activate β_2_-integrins and thus promote adhesion of PMN to the vascular endothelium. Similarly, we have found that Pyk2 is required for RelA/p65 activation and nuclear translocation to cause EC inflammation [33]. Taken together, these findings are consistent with the possibility that nmMLCK may engage the same signaling pathway to induce the expression of ICAM-1 in EC and activation of β_2_ integrins in PMN in order to promote EC-PMN interactions occurring in ALI (Fig. 7).

In summary, this study establishes an important role of nmMLCK in the mechanism of lung vascular inflammation associated with intravascular coagulation by its ability to promote nuclear localization and transcriptional activity of RelA/p65 and consequently, expression of proinflammatory genes in vascular endothelium. These data may also explain the effect of recombinant activated protein C (APC) in ameliorating vascular inflammation and improving survival in mice primarily by impairing thrombin/PAR-1 signaling [51], [52]. Additionally, our findings raise the possibility that attenuation of endotoxin-induced vascular inflammation and lethality seen in transgenic mice that express thrombomodulin (TM) lacking its extracellular lectin-like domain (TM^LeD/Led^) [53] may involve impaired nmMLCK signaling. Thus, this study further underscores the importance of targeting nmMLCK as an effective therapeutic strategy to control inflammatory lung injury.
